# Ivermectin Attenuates CCl_4_-Induced Liver Fibrosis in Mice by Suppressing Hepatic Stellate Cell Activation

**DOI:** 10.3390/ijms232416043

**Published:** 2022-12-16

**Authors:** Hanglu Ying, Long Li, Yufen Zhao, Feng Ni

**Affiliations:** 1 Institute of Drug Discovery Technology, Ningbo University, Ningbo 315211, China; 2 Qian Xuesen Collaborative Research Center of Astrochemistry and Space Life Sciences, Ningbo University, Ningbo 315211, China

**Keywords:** ivermectin, liver fibrosis, hepatic stellate cells, TGF-β1, inflammation

## Abstract

Liver fibrosis, a common liver dysfunction with high morbidity and mortality rates, is the leading cause of cirrhosis and hepatocellular carcinoma, for which there are no effective therapies. Ivermectin is an antiparasitic drug that also has been showing therapeutic actions in many other diseases, including antiviral and anticancer actions, as well as treating metabolic diseases. Herein, we evaluated the function of ivermectin in regulating liver fibrosis. Firstly, carbon tetrachloride (CCl_4_)-injected Balb/c mice were used to assess the antifibrosis effects of ivermectin in vivo. Further, CFSC, a rat hepatic stellate cell (HSC) line, was used to explore the function of ivermectin in HSC activation in vitro. The in vivo data showed that ivermectin administration alleviated histopathological changes, improved liver function, reduced collagen deposition, and downregulated the expression of profibrotic genes. Mechanistically, the ivermectin treatment inhibited intrahepatic macrophage accumulation and suppressed the production of proinflammatory factors. Importantly, the ivermectin administration significantly decreased the protein levels of α-smooth muscle actin (α-SMA) both in vivo and in vitro, suggesting that the antifibrotic effects of ivermectin are mainly due to the promotion of HSC deactivation. The present study demonstrates that ivermectin may be a potential therapeutic agent for the prevention of hepatic fibrosis.

## 1. Introduction

Liver fibrosis is a type of liver disorder sourced from a variety of chronic liver diseases, including nonalcoholic fatty liver disease (NAFLD), alcoholic liver disease, hepatitis B or C virus, and hepatic toxic damage [1,2]. It is mainly characterized by excessive collagen deposition associated with chronic liver injury and could progress to cirrhosis or hepatocellular carcinoma [3,4]. Currently, hepatic stellate cell (HSC) activation has been identified as the primary mechanism for the initiation and progression of hepatic fibrosis, while activated HSCs could convert into myofibroblasts and produce numerous extracellular matrix proteins [5,6]. As a worldwide health problem, there is still no effective agent for the treatment of liver fibrosis [7]. Therefore, it is necessary to develop novel therapies for the prevention of liver fibrosis [8].

The avermectins are a class of macrocyclic lactones, which can be naturally produced by *Streptomyces avermitilis* [9]. Ivermectin, a derivative of avermectin, is synthesized via the double-bond hydrogenation of avermectin at the C22–C23 positions, which are mainly composed of two components, 22,23-dihydroavermectin B1a (≥80%) and 22,23-dihydroavermectin B1b (≤20%) [10]. As an FDA-approved broad-spectrum anthelmintic agent, ivermectin is one of the most widely used antiparasitic drugs in veterinary and human medicine against many parasitic diseases, including onchocerciasis, lymphatic filariasis, strongyloidiasis, and trichinellosis [11,12]. In addition to its antiparasitic effect, ivermectin has also been reported to have a variety of other pharmacological functions against viral infection, cancer, asthma, and metabolic diseases [13,14,15]. The antivirus effects of ivermectin have been found in various flaviviruses, including yellow fever virus, tick-borne encephalitis virus, and Japanese encephalitis virus [10]. Interestingly, ivermectin also exhibits antiviral activity against SARS-CoV-2, the coronavirus that causes coronavirus disease 2019 (COVID-19) [16]. The inhibition of NS3 helicase or the suppression of the importin α/β1-mediated nuclear import of viral proteins may be the antiviral mechanism of ivermectin [17,18]. Numerous studies have revealed the anticancer effects of ivermectin in a variety of cancers, such as leukemia, esophageal squamous cell carcinoma, melanoma, and breast cancer [19,20,21,22]. A pharmacological study in a mouse model of allergic asthma revealed the anti-inflammatory activities of ivermectin, suggesting that this compound may play a protective role in diseases closely related to inflammation [13]. As the progression of various liver diseases, including NAFLD, hepatic insulin resistance, and liver fibrosis, is closely related to the inflammatory response, ivermectin might play a beneficial role in liver diseases [23]. It has been reported that ivermectin could regulate glucose and lipid metabolism and has therapeutic effects on NAFLD. However, it is still unclear whether it affects the development of liver fibrosis [24,25].

In this study, we investigated the function and potential mechanisms of ivermectin in liver fibrosis in CCl_4_-induced fibrotic model mice and in transforming growth factor β1 (TGF-β1)-stimulated HSCs.

## 2. Results

### 2.1. Ivermectin Ameliorated CCl_4_-Induced Liver Injury in Mice

**Figure 1:**
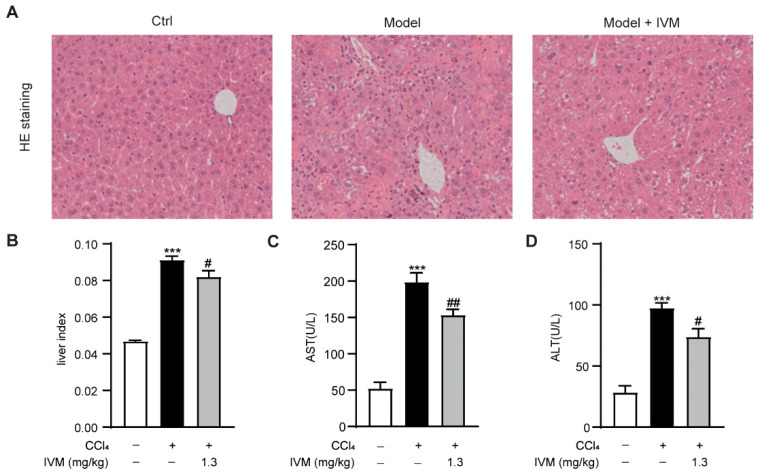
Effects of ivermectin on CCl_4_-induced hepatic pathological and biochemical parameters in mice: (**A**) liver tissues were stained with hematoxylin and eosin (HE) for the histopathological analysis (original magnifications, ×200); (**B**) liver index values; (**C**,**D**) plasma AST and ALT levels. Data are expressed as means ± SEM (*n* = 6–8/group). Note: *** *p* < 0.001 vs. control group, # *p* < 0.05, ## *p* < 0.01 vs. CCl_4_-treated model group.

To explore the contribution of ivermectin to liver fibrosis, we first examined the protective effects of ivermectin on liver injury in the CCl_4_-induced hepatic fibrosis model in mice. As shown in Figure 1, the CCl_4_ injection caused severe architectural changes in Balb/c mice, while the ivermectin administration significantly attenuated these histopathological changes (Figure 1A). Meanwhile, the CCl_4_-triggered upregulation of the liver index was markedly decreased after ivermectin treatment (Figure 1B). In addition, a significant elevation of the activity levels of aspartate aminotransferase (AST) and alanine aminotransferase (ALT) was observed in the CCl_4_-treated mice. However, the ivermectin obviously reduced the increases in AST and ALT (Figure 1C,D). These results demonstrate that ivermectin could protect mice against CCl_4_-induced liver injury.

### 2.2. Ivermectin Inhibits CCl_4_-Induced Fibrogenesis in Mice

**Figure 2:**
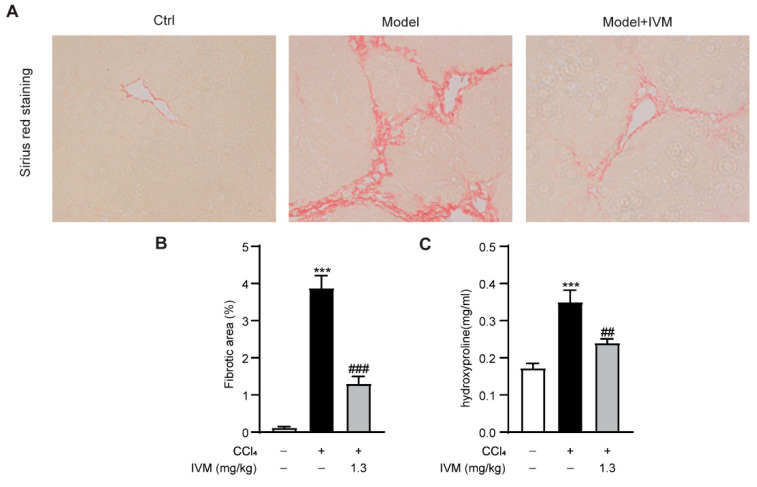
Ivermectin inhibited fibrosis in CCl_4_-treated mice: (**A**) Sirius red staining (original magnification, ×200) of liver tissues; (**B**) quantitation of fibrotic areas in the liver; (**C**) hydroxyproline levels in the liver. Data are expressed as means ± SEM (*n* = 6–8/group). Note: *** *p* < 0.001 vs. control group, ## *p* < 0.01, ### *p* < 0.001 vs. CCl_4_-treated model group.

To investigate whether ivermectin could alleviate liver fibrosis in vivo, the collagen accumulation in the liver from sacrificed mice was detected via Sirius red staining. The results showed that the collagen deposition was significantly increased, while the ivermectin treatment led to a decrease in the fibrotic area (Figure 2A,B). In accordance with the collagen staining results, the ivermectin also greatly decreased the hepatic hydroxyproline levels in CCl_4_-induced fibrosis mice (Figure 2C).

### 2.3. Ivermectin Suppresses Inflammation

**Figure 3:**
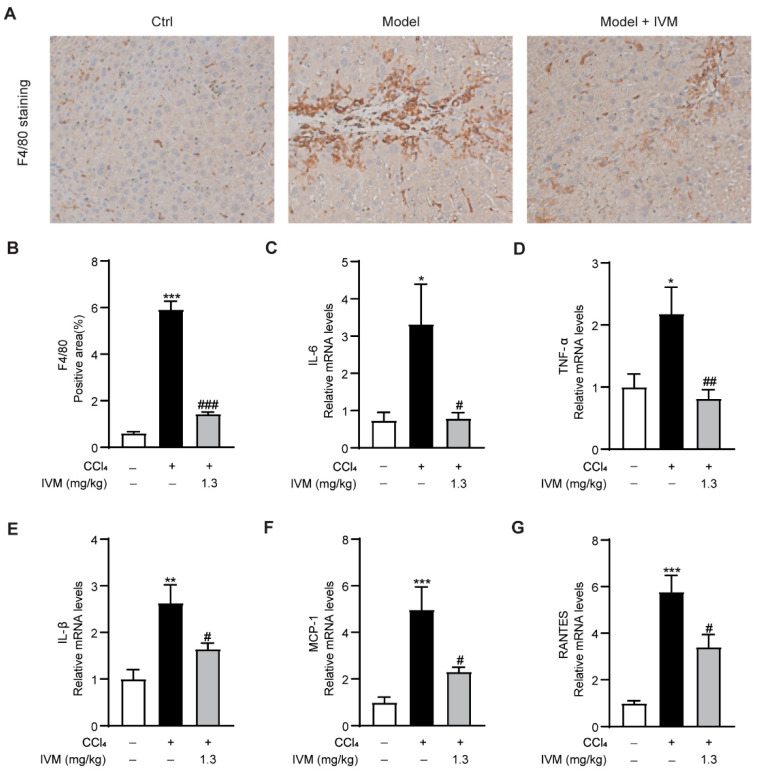
Anti-inflammatory effects of ivermectin in liver tissues from mice who received a CCl_4_ injection: (**A**) hepatic F4/80 detection via immunohistochemistry (IHC) staining (original magnification, ×200) in liver tissues; (**B**) F4/80-positive area; (**C**–**G**) mRNA expression levels of IL-6, TNF-α, IL-1β, MCP-1, and RANTES in liver tissues. Data are expressed as means ± SEM (*n* = 6–8/group). Note: * *p* < 0.05, ** *p* < 0.01, *** *p* < 0.001 vs. control group, # *p* < 0.05, ## *p* < 0.01, ### *p* < 0.001 vs. CCl_4_-treated model group.

Intrahepatic macrophage and various inflammatory factors also play a key role in the development of liver fibrosis [26]. To ascertain whether ivermectin treatment affects inflammation in CCl_4_-injected mice, macrophage accumulation and the expression of proinflammatory cytokines were detected. The results showed that in the livers of CCl_4_ model mice, F4/80, the biomarker of activated macrophages, was markedly increased, whereas an obvious decrease was detected in mice that received the ivermectin treatment (Figure 3A,B). Moreover, the CCl_4_-treated mice showed significant increases in the expression of interleukin 6 (IL-6), tumor necrosis factor α (TNF-α), interleukin-1β (IL-1β), monocyte chemoattractant protein-1 (MCP-1), and RANTES (regulated upon activation, normal T-cell expressed and secreted) compared to control mice. However, the levels of the above inflammatory mediators were obviously downregulated in ivermectin-treated mice (Figure 3C–G). Collectively, these data indicated that the inflammatory response in CCl_4_ model mice is suppressed after ivermectin treatment.

### 2.4. Ivermectin Regulates the Expression of Fibrotic Genes

**Figure 4:**
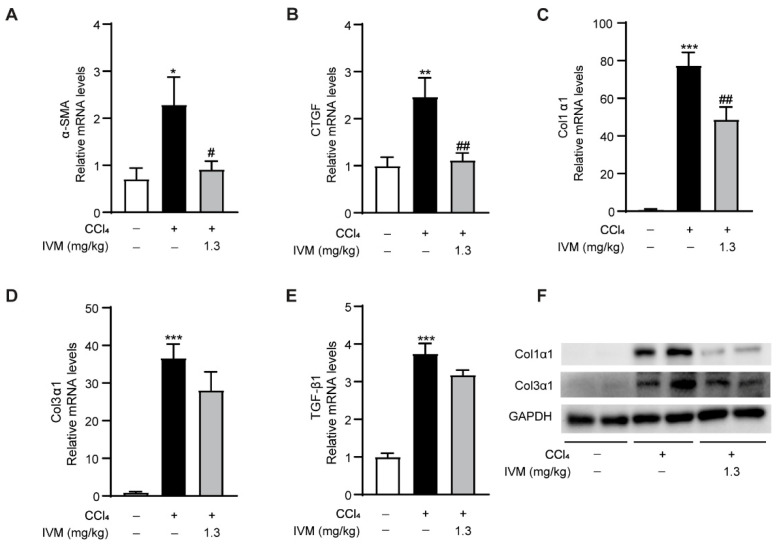
Impacts of ivermectin on the CCl_4_-induced elevation of profibrogenic genes in the liver: (**A**–**E**) mRNA expression levels of α-SMA, CTGF, Col1α1, Col3α1, and TGF-β1 in liver tissues; (**F**) protein expression levels of Col1α1 and Col3α1 in the liver. Data are expressed as means ± SEM (*n* = 6–8/group). Note: * *p* < 0.05, ** *p* < 0.01, *** *p* < 0.001 vs. control group, # *p* < 0.05, ## *p* < 0.01 vs. CCl_4_-treated model group.

To explore whether fibrosis inhibition in ivermectin-treated mice was associated with the modulation of fibrotic genes, the mRNA expression levels of α-smooth muscle actin (α-SMA), connective tissue growth factor (CTGF), collagen type 1 α 1 (Col1α1), collagen type 3 α 1 (Col3α1), and TGF-β1 were examined. As shown in Figure 4, all of the above fibrosis-related genes were upregulated in CCl_4_-injected mice. Interestingly, α-SMA, CTGF, and Col1α1 were markedly downregulated after the ivermectin treatment, while the expression levels of Col3α1 and TGF-β1 were also reduced but with less significance (Figure 4A–E). Furthermore, the Western blot results suggested that the Col1α1 and Col3α1 levels were elevated in CCl_4_-injected mice and were decreased by the ivermectin treatment, which validated the real-time PCR data to some extent (Figure 4F).

### 2.5. Ivermectin Inhibits HSC Activation in the Livers of CCl_4_-Treated Mice

**Figure 5:**
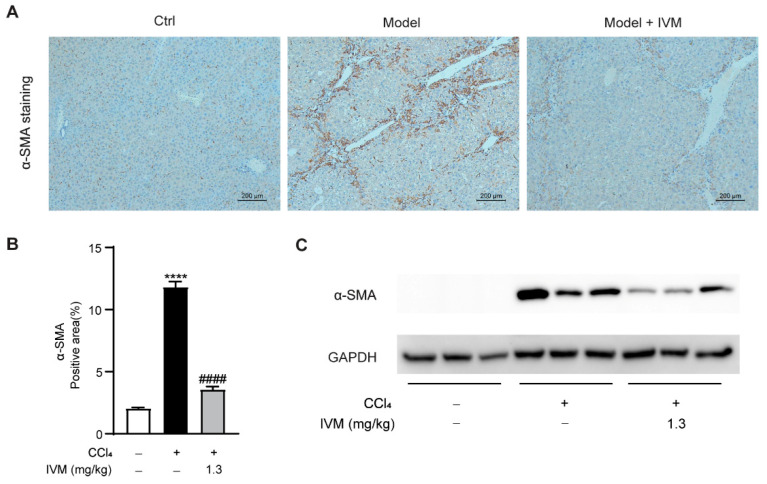
Effects of ivermectin on HSC activation in the liver in CCl_4_-treated mice: (**A**) IHC staining of α-SMA in the liver; (**B**) α-SMA-positive areas; (**C**) the Western blot analysis of α-SMA protein expression in the liver. Data are expressed as means ± SEM (*n* = 6–8/group). Note: **** *p* < 0.0001 vs. control group, #### *p* < 0.0001 vs. CCl_4_-treated model group.

To evaluate the change of the HSC phenotype in the liver, the expression of hepatic α-SMA was detected using IHC staining and Western blotting. As shown in Figure 5, the CCl_4_-induced model mice treated with ivermectin exhibited significantly decreased α-SMA expression compared to those mice only treated with the vehicle. These data suggest that ivermectin inhibits HSC activation in the context of CCl_4_-induced liver fibrosis.

### 2.6. Effects of Ivermectin on Cell Viability in CFSC Cells

**Figure 6:**
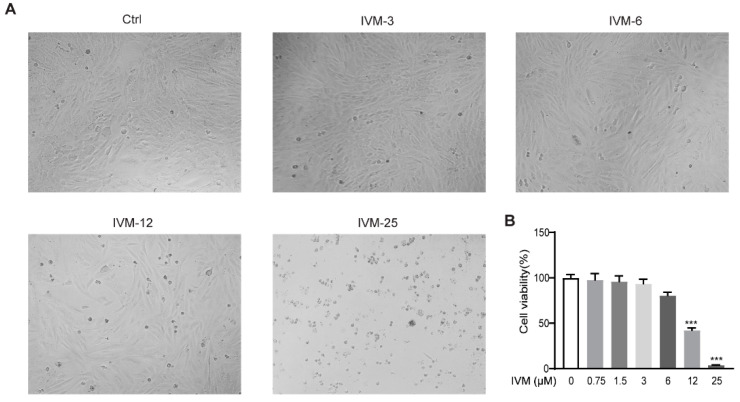
Impacts of ivermectin on cell viability in CFSC cells: (**A**) bright-field images of CFSC cells; (**B**) cell viability of CFSC cells assessed using a cell counting kit-8 (CCK-8) assay. Data are expressed as means ± SEM (*n* = 3). Note: *** *p* < 0.001.

To assess the cytotoxic effects of ivermectin on HSCs, CFSC (a rat HSC cell line) cells were incubated with growing doses of ivermectin (3, 6, 12, and 25 μM) or vehicle. The results showed that the low-dose ivermectin treatment (3 and 6 μM) had no significant effect on the cell survival, whereas the high-dose ivermectin treatment (12 and 25 μM) obviously reduced the cell viability (Figure 6).

### 2.7. Ivermectin Suppresses TGF-β1-Induced HSC Activation In Vitro

**Figure 7:**
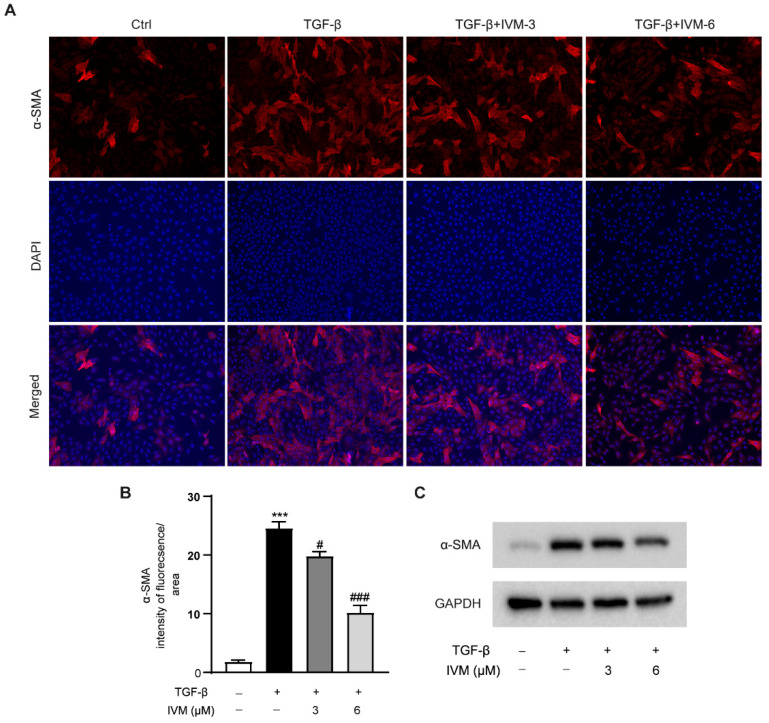
Effects of ivermectin on TGF-β1-induced HSC activation: (**A**) IHC staining of α-SMA in CFSC cells that received TGF-β1 stimulation and ivermectin treatment; (**B**) α-SMA intensity of fluorescence; (**C**) the Western blot analysis of α-SMA protein expression in CFSC cells. Data are expressed as means ± SEM (*n* = 3). Note: *** *p* < 0.001 vs. control group, # *p* < 0.05, ### *p* < 0.001 vs. TGF-β1-treated model group.

To further investigate whether ivermectin could directly regulate HSC activation, TGF-β1-stimulated CFSC cells were used in this study. As shown in Figure 7, the α-SMA expression was progressively upregulated in CFSC cells upon TGF-β1 stimulation, suggesting the activation of HSCs in vitro. However, the ivermectin pretreatment dose-dependently reduced the TGF-β1-induced elevation of the α-SMA protein levels, revealing that ivermectin could suppress TGF-β1-induced HSC activation in vitro.

## 3. Discussion

William C. Campbell and Satoshi Ōmura were awarded the 2015 Nobel Prize in Physiology and Medicine for their discoveries leading to ivermectin [27]. Ivermectin is a broad-spectrum antiparasitic drug against various nematodes and ectoparasites [15]. Recently, the pharmacological application of ivermectin has been extended to the treatment of multiple diseases, including cancer, inflammation, and viral infection [28,29,30]. Some previous reports have revealed that ivermectin displays glucose- and cholesterol-reducing, insulin-resistance-improving, and fatty-liver-ameliorating properties in rodents [14,24]. Our present study reveals a previously unrecognized crucial role of ivermectin in attenuating liver fibrosis through suppressing HSC activation. In this study, we found that ivermectin attenuated liver injury, reduced plasma levels of transaminase, suppressed hepatic accumulation of macrophages, inhibited the production of proinflammatory factors, and alleviated the expression of fibrotic genes. All of the above data demonstrated the beneficial effects of ivermectin on liver fibrosis.

As one of the most commonly used inducers of liver fibrosis, CCl_4_ has hepatotoxicity and can cause hepatocyte injury [31]. In our present study, severe liver damage was observed in mice exposed to repeated CCl_4_ injection, as evidenced by the presence of parenchymal necrotic zones, inflammatory cell infiltration, and elevated plasma AST and ALT activities. To our delight, the ivermectin treatment greatly alleviated hepatic histopathological changes in parallel with the improvement in liver function. Previous studies have found that a number of fibrogenesis-associated genes are involved in the occurrence and development of hepatic fibrosis [32,33]. Among these genes, α-SMA is the most classic biomarker of HSCs activation; CTGF could stimulate the production of extracellular matrix (ECM) components, while Col1a1 is the most important component of the ECM [34,35,36]. Our data revealed that ivermectin administration significantly reduced α-SMA, CTGF, and Col1α1 expression in CCl_4_-injected hepatic tissues.

Numerous studies have reported that HSC activation is the key driving factor for the progression of liver fibrosis [37,38]. In the present study, ivermectin significantly reduced hepatic α-SMA expression in mice injected with CCl_4_. The activation of HSC transdifferentiation in the liver is mainly due to the stimulation of TGF-β1, which is a member of the TGF-β superfamily and could be synthesized and released from various cell types during acute and chronic liver injury [39,40]. As a key regulator of liver physiology and pathology, the TGF-β1 signaling pathway is considered to be an important target for the treatment of liver fibrosis [41,42]. However, the effect of ivermectin on the expression of TGF-β1 was not significant in CCl_4_-induced hepatic fibrosis mice in the present study, suggesting that the antifibrotic function of ivermectin may be independent of the regulation of TGF-β1 production. Interestingly, our in vitro study showed that ivermectin could obviously decrease the protein levels of α-SMA in TGF-β1-stimulated CFSC cells. These data imply that ivermectin attenuates hepatic fibrosis by suppressing HSC activation.

Ivermectin is known as a macrocyclic lactone with an antiparasitic function [43]. The macrocyclic lactones include two subfamilies, avermectins and milbemycins [44]. In addition to ivermectin, the avermectins also include abamectin, doramectin, selamectin, and eprinomectin, while the milbemycins include milbemycin oxime and moxidectin [45]. These antiparasitics have a similar structure, containing a 16-membered lactone ring [46,47,48]. To investigate which compound exerts the best anti fibrotic activity, we compared the regulatory effects of ivermectin, abamectin, doramectin, selamectin, moxidectin, and eprinomectin on the activation of HSCs, and found that ivermectin had the most significant inhibitory effect on the expression of α-SMA in TGF-β1-treated CFSC cells, indicating that among these macrocyclic lactones, ivermectin should be the most effective against liver fibrosis (Figure S1).

Farnesoid X receptor (FXR) is a nuclear receptor that plays a key role in the modulation of lipids, bile acid, cholesterol, and the glucose metabolism [49,50]. A previous study identified ivermectin as a ligand for FXR and revealed its function in regulating cholesterol and glucose homeostasis in an FXR-dependent manner [14]. The nuclear receptor small heterodimer partner (SHP) is an inductive target of FXR [51,52]. It has been reported that SHP mediated the protective effect of FXR against liver fibrosis through suppressing HSC activation [53]. To explore whether the FXR/SHP signaling pathway mediated the beneficial effect of ivermectin against hepatic fibrosis, we assessed the hepatic expression of SHP in CCl_4_-treated mice and found that the CCl_4_ injection significantly reduced the SHP mRNA expression. However, the ivermectin administration failed to elevate the expression of SHP in the liver (Figure S2). Our present data indicated that the protective effect of ivermectin on hepatic fibrosis may be independent of the FXR signaling pathway.

The accumulating evidence suggests that inflammation could drive the progression of liver fibrosis [54,55]. An intrahepatic macrophage is closely related to the initiation and development of liver fibrosis, which could be activated in the case of liver injury and could further promote the expression and secretion of proinflammatory cytokines, such as IL-6, TNF-α, and IL-1β, triggering inflammatory responses in the liver [56,57]. Consistent with previous studies, this study also found that a CCl_4_ injection in mice significantly increased the hepatic expression of chemokines including MCP-1 and RANTES, which could recruit more immune cells into the liver, aggravating the progression of liver fibrosis [58,59]. Ivermectin has been reported to inhibit the infiltration of inflammatory cells and the expression of cytokines in asthmatic mice [13]. In addition, ivermectin has been shown to increase the survival rate of lipopolysaccharide-treated mice by suppressing the production of inflammatory cytokines [60]. Importantly, our present study found that ivermectin inhibited the number of F4/80-positive macrophages and decreased the expression levels of IL-6, TNF-α, IL-1β, MCP-1, and RANTES in CCl_4_-treated mice. These results indicated that the anti-inflammatory effect of ivermectin may also contribute to its alleviation of liver fibrosis.

Together, we provide evidence that ivermectin alleviated CCl_4_-induced liver injury, inflammation, and fibrogenesis in mice. The inhibition of TGF-β1-stimulated HSC activation is the main mechanism underlying the protective effects of ivermectin against liver fibrosis. These findings demonstrate that ivermectin, which is an FDA-approved drug, maybe a potential pharmacological agent for the therapy of hepatic fibrosis.

## 4. Materials and Methods

### 4.1. Animal Experiments

The Balb/c male mice were 6–8 weeks of age and were acquired from Vital River Laboratory Animal Technology Co., Ltd. (Beijing, China). The Animal Ethics and Welfare Committee of Ningbo University approved all animal experiments (Approval No. NBU20220121). The mice were housed at a controlled temperature range of 20–23 °C and 50–60% humidity with 12 h light/12 h dark cycles and ad libitum access to food and water. The mice in the control group only received intraperitoneal injections of olive oil (Sangon Biotech, Shanghai, China). The liver fibrosis model was established via CCl_4_ (Macklin, Shanghai, China) injection (1 mL/kg body weight, i.p., dissolved in olive oil) for 4 weeks, twice a week, as previously reported [61]. A subset of model mice received daily intraperitoneal injections of ivermectin (1.3 mg/kg; Macklin, Shanghai, China) or vehicle during the period of liver fibrosis modeling.

### 4.2. Cell Culture

CFSC, a rat HSC cell line, was grown in DMEM supplemented with 10% FBS. The cells were maintained in a cell incubator with 5% CO_2_ under 37 °C. For the cell viability assay, the CFSC cells were treated with ivermectin (3 μM, 6 μM, 12 μM, 25 μM) for 48 h. For the HSC activation assay, the CFSC cells were pretreated with ivermectin at concentrations of 3 μM and 6 μM for 1 h and then treated with TGFβ1 (10 ng/mL; Novoprotein, Shanghai, China) for another 48 h.

### 4.3. Histological Assessments

The liver tissues were fixed in a 4% paraformaldehyde (PFA) solution (Solarbio, Beijing, China) for 24 h at 4 °C and then embedded in paraffin (Sigma Aldrich, Shanghai, China). The paraffin-embedded liver tissues were sectioned (5 μm) and stained with HE to evaluate histological changes or stained with Sirius red to assess collagen deposition following standard procedures.

### 4.4. Immunofluorescence (IF) Analysis

The CFSC cells were fixed with 4% PFA and stained with the mouse anti-α-SMA antibody (Sigma Aldrich, Shanghai, China), then stained with the CoraLite594-conjugated goat anti-mouse IgG (Proteintech, Wuhan, China). The nuclei were counterstained with DAPI (Solarbio, Beijing, China). A Zeiss Axio Observer 5 microscope (Carl Zeiss, Oberkochen, Germany) was used for the observations.

### 4.5. Biochemistry Assay

The levels of AST, ALT, and hydroxyproline were determined using colorimetric assay kits (Nanjing Jiancheng Bioengineering Institute, Nanjing, China) according to the manufacturer’s protocols.

### 4.6. IHC Assay

The harvested liver tissue samples were fixed in 4% PFA, embedded in paraffin, and sectioned to 5 μm for immunohistochemistry. The sections were deparaffinized in xylene and rehydrated in graded alcohol. Next, antigen retrieval was carried out by heating sections in citrate buffer for 10 min. Then, the sections were incubated with 0.3% H_2_O_2_ for 10 min to block the endogenous peroxidase activity. After blocking with 5% BSA, the anti-F4/80 antibody (Abcam, Shanghai, China) or anti-α-SMA antibody (Sigma Aldrich, Shanghai, China) was incubated overnight at 4 °C, followed by incubation with HRP-conjugated secondary antibodies (Proteintech, Wuhan, China) for 1 h. Finally, the sections were stained with the DAB substrate solution and co-stained with hematoxylin.

### 4.7. Western Blot Analysis

The protein extracts from liver tissues and CFSC cells were prepared in RIPA lysis buffer (Beyotime Biotechnology, Shanghai, China) supplemented with protease inhibitor cocktails (Roche, Shanghai, China). Then, the protein samples were separated using SDS-PAGE and transferred to PVDF membranes (Merck Millipore, Shanghai, China). After blocking with 5% skim milk, the membranes were incubated with mouse anti-α-SMA antibody (Sigma Aldrich, Shanghai, China) or rabbit anti-GAPDH antibody (Proteintech, Wuhan, China) overnight at 4 °C and subsequently incubated with HRP-conjugated goat anti-mouse IgG or HRP-conjugated goat anti-rabbit IgG (Proteintech, Wuhan, China) for 2 h at room temperature. Finally, the bands were examined with an ECL assay kit (NCM Biotech, Suzhou, China).

### 4.8. RNA Extraction, cDNA Synthesis, Real-Time PCR

The total RNA was isolated from liver tissues using a Total RNA Kit (Omega Bio-Tek, Norcross, GA, USA). The preparation of the cDNA was carried out with a FastQuant cDNA kit (Tiangen, Beijing, China). The real-time PCR was carried out with SuperReal PreMix Plus (SYBR Green) (Tiangen, Beijing, China) for the quantification of target gene expression and normalized to the expression of GAPDH using 2^−ΔΔ^Ct method.

### 4.9. Statistical Analyses

The statistical analyses were performed with a one-way analysis of variance (ANOVA) followed by the Tukey multiple comparison test in GraphPad prism 8.3.0 (GraphPad Software, La Jolla, CA, USA). The data are expressed as the means ± the standard error of the mean (SEM). Statistical significance was set as *p* < 0.05.

## Data Availability

All data generated for this research are included in the article or supplementary materials.

## Supplementary Materials

The following supporting information can be downloaded at: https://www.mdpi.com/article/10.3390/ijms232416043/s1.
