# Supplementary material for: Ivermectin Attenuates CCl4-Induced Liver Fibrosis in Mice by Suppressing Hepatic Stellate Cell Activation
Source: Int J Mol Sci. 2022 Dec 16;23(24):16043. doi: 10.3390/ijms232416043 (PMC9782196; doi:10.3390/ijms232416043)
Supplement: Supplementary file 1 [file ijms-23-16043-s001.zip › ijms-1964121-supplementary.pdf]

Supplementary Material

Supplementary Figure S1

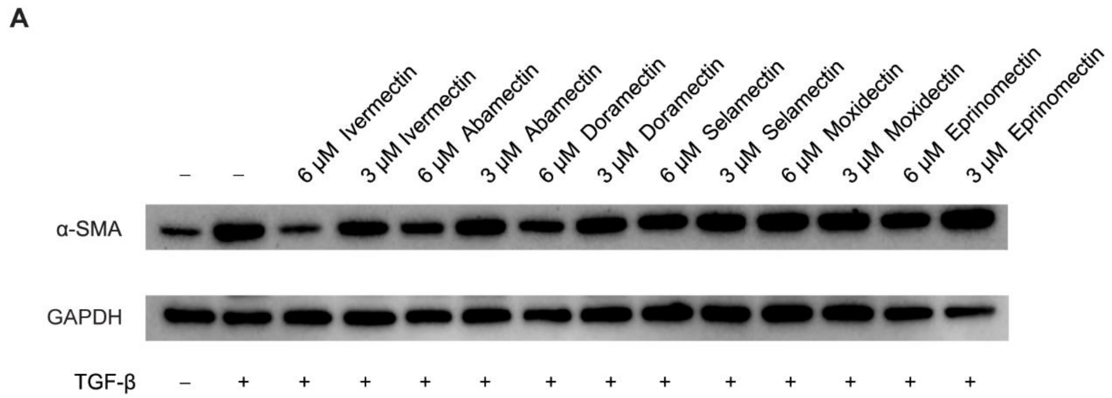

Figure S1. The protein levels of α-SMA in CFSC cells after TGF-β1 stimulation and macrocyclic lactones treatment. Western blot analyzed α-SMA protein levels in TGF-β1-stimulated CFSC cells treated with different doses (6 μM, 3 μM) of ivermectin, abamectin, doramectin, selamectin, moxidectin, and eprinomectin.

Supplementary Figure S2

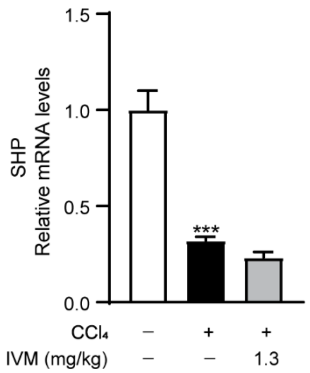

Figure S2. The mRNA expression of SHP in liver after Ccl4 injection and ivermectin treatment. Hepatic SHP expression in mice analyzed by Real-time PCR. Data represent the mean ± SEM. *** $P < 0.001$ vs vehicle-treated control group.
